# Disclosure in the era of generative artificial intelligence

**DOI:** 10.3389/fdgth.2026.1826808

**Published:** 2026-04-23

**Authors:** Khalifah A. Aldawsari

**Affiliations:** Department of Pediatrics, Section of Cardiology, University of Colorado, Aurora, CO, United States

**Keywords:** disclosure, generative artificial intelligence, large language models, research integrity, scientific writing

## Abstract

Generative artificial intelligence (AI) has rapidly become embedded in academic writing, assisting with tasks ranging from language editing to drafting text and producing evidence. Despite the wide range of AI use, the expectations for disclosure remain inconsistent. Several journals use binary disclosure statements that fail to distinguish minor language assistance from uses that have a significant impact on the manuscript structure and data interpretation. This article proposes a risk-proportional approach separating disclosure by risk.

## 1 Introduction

Generative artificial intelligence (AI) has moved quickly from novelty to routine use in academic writing (1). Large language models now assist with outlining manuscripts, refining prose, summarizing literature, and generating first-pass text. What began as simple spelling correction has expanded into broad assistance that has an impact on almost every stage of scientific work. Recent evidence suggests that adoption is widespread. An early 2023 study found that approximately 48% of the submitted manuscripts contained AI-assisted writing in more than 10% of the text (1).

The debate is no longer about whether AI is used in academic writing. It clearly is. The more pressing issue is how its use should be disclosed in a way that protects scientific quality without discouraging reasonable, and responsible use.

Despite the increased adoption of AI, transparency guidelines have lagged behind with inconsistent disclosure policies across journals (2, 3). A simple checkbox at the time of submission for AI use does not account for the nuanced ways AI is utilized. This commentary introduces a risk-based framework to promote more transparent and proportionate reporting by authors.

## 2 Benefits and risks of binary disclosure

As in many areas of medicine, AI use ranges from simple to more complex tasks. At one end, it assists with spelling and grammar correction and improving clarity. On the other end, it takes on higher-risk tasks that directly impact study results, such as transforming data, comparing studies, and drawing conclusions. Treating all uses the same obscures the difference between basic language support and substantive intellectual contribution.

For many researchers who write in a second language, these tools offer clear benefits (4). Language barriers can negatively affect the publication of otherwise strong science. AI-assisted editing can improve clarity and organization, allowing ideas to be judged on their merit rather than language and fluency (5). In this regard, AI use may promote equity by lowering linguistic barriers to publication and allowing the dissemination of global knowledge (6). It can also reduce time and cost spent on drafting tasks, enabling investigators to focus on study design, analysis, and interpretation (7).

Having said that, the risks are equally substantial. AI has been noted to generate facts, provide interpretations with a false sense of assurance, and cite false references (8). In addition, when tasked with summarizing the literature, some studies may be omitted without a clear rationale, leading to unreliable results.

Another important concern with AI use is the risk of breaking confidentiality, which requires careful oversight. This risk has become more relevant with the recent introduction of built-in data analysis features in AI tools, such as ChatGPT, which can quickly analyze large datasets and produce statistical summaries and visualizations. Understandably, these features can be appealing to researchers due to their fast turnaround and convenience, especially when faced with an approaching deadline. However, uploading protected health information to external AI platforms violates HIPAA regulations and should never occur, as protecting identifiable patient data must remain a fundamental responsibility of all investigators.

For these reasons, AI cannot assume accountability for the accuracy, ethics, or integrity of the scientific work. Authors, not algorithms, bear full responsibility for every statement, citation, and conclusion. Disclosure practices should reflect this principle.

## 3 Proportional risk disclosure

A practical solution is to separate the two distinct obligations: disclosure to editors and disclosure to readers. Disclosure to editors should be routine and comprehensive with no exceptions. At submission, authors should describe how AI tools were used so that journals can assess the reliability of the work.

Disclosure to readers in the manuscript should be proportional to risk. The risk is defined by the likelihood that AI use could influence the scientific material. When AI use is limited to language editing, such as grammar or spelling checks, mandatory statements may add little value and may unintentionally disadvantage those relying on such tools for language support. In contrast, when AI is used to generate scientific content, perform statistical analysis, or form an interpretation, transparent and detailed disclosure becomes essential. Readers should be able to understand how conclusions were produced and what processes influenced the final results to enable reproducibility.

Several journals have now established standards for disclosing AI use (9). The simple yes or no disclosure was acceptable in the early phase, when AI played a limited role in writing. However, as AI becomes more integrated into academic research, authors would benefit from clearer practical guidance rather than broad cautions.

**Figure 1:**
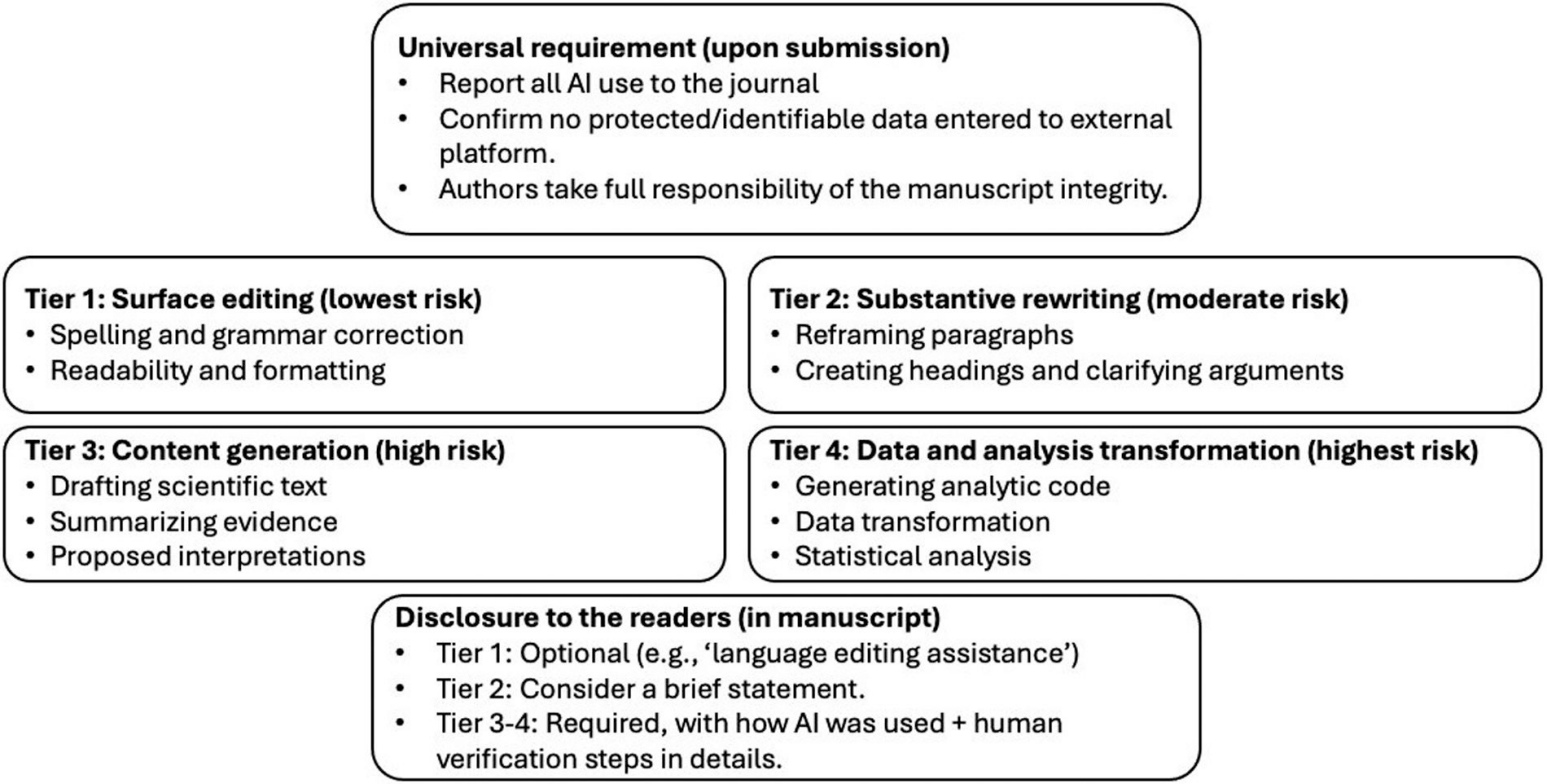
DISCLOSE-AI: risk-proportional disclosure framework for generative AI use in scientific writing.

To address this gap, I propose a framework, DISCLOSE-AI, that matches disclosure with the level of risk (Figure 1). The framework has four tiers. If AI is only used for grammar and spelling, public disclosure in the manuscript may not be required. If it is used for major writing or drawing conclusions, this should be clearly described in the manuscript. Nonetheless, all use of AI should be reported to the journal when submitting, regardless of the tier level. In addition, editor-level disclosure should remain accessible to readers upon request. In cases of hybrid use across multiple tiers, readers disclosure should reflect the highest tier.

This approach preserves transparency where it matters most and recognizes that minor language editing is now common.

Importantly, disclosure should not be punitive. A recent study reported that individuals who use AI were subjected to negative social perception and lower competency evaluation (10). The goal of disclosure, therefore, is not to stigmatize AI use but to clarify accountability. When used thoughtfully, AI can improve the manuscript quality (11). We are still early in bringing these tools into academic practice, and disclosure standards will change as more evidence becomes available. Still, authors should always take full responsibility for their work. Scientific credibility depends not just on results, but also on how those results are achieved.

## Data Availability

The original contributions presented in the study are included in the article, further inquiries can be directed to the corresponding author.
